# Profile of self-harming patients admitted in a South African hospital

**DOI:** 10.4102/jcmsa.v3i1.114

**Published:** 2025-04-30

**Authors:** Mohale V. Senyolo, Indiran Govender, Tombo Bongongo

**Affiliations:** 1 Department of Family Medicine and Primary Health Care, School of Medicine, Sefako Makgatho Health Sciences University, Pretoria, South Africa

**Keywords:** profile, patients, self-harm, Dr George Mukhari Academic Hospital, Pretoria, South Africa

## Abstract

**Background:**

Self-harm, as intentionally hurting oneself, has grown to be a major public health issue in recent years. Such act can be carried out without the deliberate intent to kill oneself. This study aimed to profile self-harming patients admitted to a South African hospital.

**Methods:**

A cross-sectional design based on retrospective record review of self-harming patients between June 2022 and May 2023 at Dr George Mukhari Academic Hospital (DGMAH).

**Results:**

Out of 223 records retrieved, the participants’ mean age was 24.6 years. Their ages ranged from 13 years to 75 years. The majority were single (*n* = 198; 89.56%), unemployed (*n* = 103; 46.19%) and did not have any comorbidities (*n* = 171; 76.68%). Human immunodeficiency virus (HIV) or acquired immunodeficiency syndrome (AIDS) (*n* = 19; 8.52%) was the most common comorbidity. Overdose and poisoning (*n* = 220; 98.65%) are common methods utilised, and with high rate happened in October (*n* = 39; 17.65%). The most common reasons were interpersonal, including family conflict (69; 30.80%) and relationship issues (*n* = 61; 27.23%). Age was linked with sex, comorbidities, method and reason (*p* = 0.008). Conflict was linked with females and relationship problems with males (*p* = 0.008).

**Conclusion:**

Self-harm is common among single females under 40 years, often because of romantic and family conflicts. Age and sex influence risk, with younger females using over-the-counter medication, older females with retroviral diseases using prescriptions. Further research, including prevention, may assist in the management of the behavior.

**Contribution:**

An alarming and growing public health risk has been raised.

## Introduction

When tackling self-harm, which is wilfully injuring oneself, the researcher’s objective in this work was to look at the behaviour by evaluating individuals who required attention or made obvious the desire to harm themselves.^1^ Self-harm is also characterised as careless behaviour intended to attract attention and prompt a help request.^1^ It can also be described as an attempted suicide with no true intention of ending one’s life.^1^ Such behaviour is commonly addressed in South African hospitals as parasuicide.

Self-harm and suicide prevention programmes have been under increasing strain in the United Kingdom (UK) in recent years.^2^ The cost-of-living problem is one of the reasons behind this. Such behaviour has become a public health concern^2^; and when examining the prevalence of hospital presentations involving self-harm and the related healthcare expenses in England, in comparison to more prosperous regions within kingdom, it seemed that the costs were similarly greater in the region’s that were more socioeconomically challenged.^3^ This behaviour can start during an early age as demonstrated in one study in England. The feeling for self-harm can arise by the age of 12 years or 13 years, and for more than half of the sample, self-harming ideas and actions persist throughout the year. Strong school ties and safe peer interactions were linked to lower rates of self-harm.^3^

The true prevalence of self-harm has been severely underestimated for a multitude of reasons, including the fact that most research relies on hospital data and that some cases never reach the hospital’s emergency or casualty unit.^4^ Some cases have been managed by general practitioners, while others are sent to private clinics or handled discreetly by friends or family. Although there appears to be underreporting in Africa, there is ample evidence that it is a concerning global phenomenon.^4^

A review was conducted on 74 studies involving youths aged 10–25 years from 18 sub-Saharan African nations.^5^ The data include the Central, Eastern, Southern and Central geographical sub-regions of sub-Saharan Africa. The study examined the prevalence of self-harming patients.^5^ The median lifetime prevalence estimate was 10.3%, and the median 12-month prevalence estimate was 16.9%, indicating significant variation across all reported prevalence estimate ranges. Western sub-Saharan Africa yielded the highest 12-month prevalence estimates, with a median of 24%, followed by Southern sub-Sahara Africa research at 16%, and Eastern sub-Sahara Africa research at 11%.^5^

In the North west province of South Africa, when profiling self-harming cases at Brits Hospital, a 5-year retrospective chart analysis of 477 cases found that the mean age was 26.6 years, the median age was 24 years and the minimum and maximum ages were 12 years and 64 years, respectively.^6^ The majority (*n* = 257; 53.88%) were between the ages of 16 years and 25 years and included females (*n* = 297; 62.26%), Africans (*n* = 391; 82.84%), singles (*n* = 352; 73.79%), unemployed (*n* = 304; 63.7%) and those who had completed secondary school were 300 (62.9%).^6^

Different demographic characteristics have been investigated. Gender disparities in self-harm tendency have been widely recognised for a long time.^6^ This finding indicates that women were more likely than males to commit parasuicide (62.3% vs. 37.7%).^6^ This is in line with several studies, such as one conducted in South Africa’s KwaZulu-Natal area that found 72% of girls^7^ and another conducted in England that found 74% were more likely to commit suicide.^8^

A Zimbabwean quantitative analysis discovered that young male, single dependent persons with a lower level of education were the most vulnerable group.^9^ According to the KwaZulu-Natal statistics, patients who demonstrate self-harm behaviour are more likely to be aged 15–30 years.^7^ This same tendency was also noticed in Brits, who reported that the majority of patients were between the ages of 16 years and 25 years, with an average participant age of 26.6 years.^6^ A unique trend was seen in the Zimbabwe research compared to KwaZulu-Natal and Brits Hospital, where the majority of patients were between the ages of 31 years and 35 years, with the 21–25 years age group following closely.^9^

In KwaZulu-Natal province of South Africa,^7,9^ the statistics show that 74% of those who reported self-harming behaviour were single, with only 16% married in a KwaZulu-Natal study.^7^ While in Brit, South Africa, it was found that 74% of persons were single, while 19% were married.^6^ In Zimbabwe, 65% of the participants were unmarried; and from the same study, married persons with dependent children have consistently lower rates of self-harm,^9^ which may be because of a stronger sense of responsibility. However, from Zimbabwe, it was suggested that economic challenges play a role in the occurrence of self-harming behaviour.^9^

In Cape Town in South Africa, it was found that depressive symptoms were more strongly associated with self-harming behaviour than a range of proximal and distal economic factors among young men living in endemic poverty.^10^ This has significant public health implications, emphasising the significance of improving young men’s access to psychiatric treatments, with depression screening being a key component. In the province of Limpopo, South Africa, limited financial resources and a lack of assistance were similarly identified as the main factors of self-harming behaviour.^11^ To make matters worse, in addition to their financial struggles, these individuals also had to tend to their family.^11^

A quantitative study in Chegutu, Zimbabwe, focused on self-harming cases involving 169 young people. The study on teenagers aimed to uncover risk factors and preventative measures for self-harming behaviour.^2^ According to the findings, risk factors among teenagers included emotions of powerlessness, despair, alcohol and drug abuse, stressful life events and family problems. These risk factors were present in 39% of all individuals. This study also discovered some protective factors associated with self-harming behaviour, including religious convictions and peer and other social support.^12^ In another study conducted in Zimbabwe, 58% of respondents in this study indicated recent divorce and grief as triggers for self-harming behaviour. Hopelessness was a factor in 50% of parasuicides, 73% had chronic low self-esteem and 73% had some sort of mental disease. In the same study, people are at a significant risk of self-harming behaviour because of a loss of hope, depression and social conditions.^9^ According to the report, 81% of respondents had disagreements with their spouses, close family members or relatives. Poor family bonds and unstable family dynamics are strong predictors of parasuicide. In this study, financial problems accounted for 73% of self-harm triggers. This lack of financial independence became an entry point for problems such as abuse and frustration, which ended in self-harming acts. In this study, a considerable proportion of respondents (54%) stated that they had demonstrated self-harming behaviour in the past. Previous history of self-harming acts and mental illness were identified as significant predictors of repeated self-harming act in a logistic regression model. As the number of risk factors increases, so does the probability of repeated self-harming act.^9^

Negative mood, induced by events or physical health difficulties, or a mix of the two, sparked self-harm ideas. This resulted in people isolating themselves. This isolation seems to amplify negative thoughts and allows them to be harboured inwardly.^13^ As individuals were left alone with their thoughts, there were limited opportunities for them to be talked to, which caused an accumulation of negative effects and self-harm ideation. With time to reflect and dwell, the first ‘trigger’ on bad mood appeared to function as a magnifying glass for the participants’ life, accentuating all their perceived challenges and unresolved issues.^13^ In the Limpopo province of South Africa, following an unstructured in-depth interview, a list of factors including unemployment, poverty and domestic violence, mental health issues like depression, other medical conditions like human immunodeficiency virus (HIV) and/or acquired immunodeficiency syndrome (AIDS) and accusations of witchcraft were established to be the motives behind self-harming behaviour.^11^

In a Kenyan study, the methods used for self-harming behaviour differ by gender, with women being more likely to use less physically damaging methods.^14^ The most popular methods were hanging, drowning and self-poisoning with organophosphates and pesticides.^14^ In terms of overdose, the most used drugs were valium, chloroquine, artane, chlorpromazine and so on. Women appear to prefer self-harming themselves via overdose.^14^ Medical medication overdose is the predominant method of self-harming oneself in developed countries. Universally common methods can be summed up as overdose, self-poisoning, drunkenness and bodily harm, hanging, drowning, jumping from heights, lying in front of a moving vehicle/train, self-inflicted injuries from cutting, shooting and piercing tools.^14^ In a Zambian study, the most common ways of self-harming included organophosphate poisoning (59%), drug overdose (32%) and acid poisoning (26%).^1^

A study in South Africa evaluated deliberate self-harm (DSH) methods among 238 tertiary hospital patients and found that self-poisoning was the most common way (80.3%), followed by prescription medicines (57.6%).^9^ Prescription pharmaceuticals, such as tricyclic antidepressants, anti-hypertensive agents and benzodiazepines, were regularly utilised by the patient, whereas over-the-counter (OTC) medications such as paracetamol, antihistamines and nonsteroidal anti-inflammatory drugs were the most used for self-harm. According to one study, 7.9% of patients ingested or inhaled poison, while 20% used prescription and OTC drugs; 14.3% engaged in body tissue injury, while 5.5% attempted hanging and self-mutilation. Male gender, a desire to flee and a history of substance misuse all raise the likelihood of employing violent DSH strategies. Men are 6.2 times more likely to adopt forceful methods, and those with a history of substance abuse are 3.3 times more likely to use violent methods.^15^At Brits Hospital in the North west province of South Africa, a study revealed a higher rate of self-harm in the summer months of January and February (13.63% and 13.42%, respectively).^6^

The South African Depression and Anxiety Group reports that 460 self-harming cases occur in the country every day.^16^ Given the self-harming statistics shown earlier in the text, the purpose of this study was to profile self-harming patients admitted to a South African hospital’s family medicine wards.

## Research methods and design

### Study design

This was a cross-sectional design based on a retrospective record review of self-harming patients admitted in family medicine wards at a South African hospital between June 2022 and May 2023.

### Study setting

The study was carried out in the family medicine wards of Dr George Mukhari Academic Hospital (DGMAH) located in Ga-Rankuwa Township, in the north of Pretoria, South Africa. It is a teaching facility for the Sefako Makgatho Health Sciences University formerly known as Medical University of Southern Africa (MEDUNSA). Dr George Mukhari Academic Hospital has two family medicine wards (one as a female ward and the other one as male ward), with a bed capacity of 36 each. Self-harming patients are admitted to these two wards; and they are supervised by specialist family physicians.

### Study population, sampling technique and sample size

All self-harming patients’ medical records registered in DGMAH’s family medicine department between 01 June 2022 and 31 May 2023 were considered for this study. The registry contains 525 medical files for the period. Only medical records that fit the study’s inclusion criteria including socio-demographic data, reasons for self-harming, admission month and methods utilised. Medical records of patients with self-harming behaviour admitted in family medicine wards from June 2022 to May 2023 were chosen for the study sample. From a population sample of 525 medical files, the sample size was computed to 223 using the Raosoft sample size calculator,^17^ with a confidence level of 95% and margin of error of 5%.

### Data collection

The researcher trained a research assistant (RA) to access medical data and decide which information to extract from the medical file based on the study’s inclusion criteria. A data-collection method created and used in a South African self-harming study in 2009^15^ and later used in the Brits study^6^ was adapted for the current study. After retrieving the medical records that met the inclusion criteria, all selected files constituted the study sample. To avoid selecting the same file several times, each file was allocated a unique number. Once a file is selected, information from the file is transmitted to the Excel spreadsheet, which serves as the data-gathering tool.

### Data analysis

Data were captured, cleaned and coded on Microsoft Excel and subsequently imported to STATA version14 for analysis. Descriptive analysis for socio-demographic variables was provided in the form of means, range and standard deviations for continuous variables as well as proportions and percentages for categorical variables using diagrams and frequency tables. Bivariate and multivariate analysis was performed to identify patterns and significant associations. Firstly, the data were stratified by age, with younger participants (≤ 18 years) and older participants (˃ 18 years). The Pearson Chi-square test of association was used to identify significant associations between age and other socio-demographic and clinical factors (*p* ≤ 0.05). A purposive selection of covariates was used to build a logistic regression model, and categorical variables were converted into numeric for multivariate analysis to see which variables would remain significant (*p* ≤ 0.05). Secondly, the data were stratified by sex to identify the patterns and associations that would be significant (*p* ≤ 0.05) and the process for bivariate and multivariate analysis was followed.

### Ethical considerations

The Sefako Makgatho Health Sciences University Research Ethics Committee (SMUREC) approved the study (No. SMUREC/M/12/2024:PG). Permission was also obtained from the Odi District Hospital authority to carry out the study in the facility. Patient confidentiality was maintained by assigning a code to each participant rather than utilising their real names in the analysis.

## Results

### Socio-demographic characteristics

The total sample included 223 patients, with a mean age of 24.6 years. The participants’ ages ranged from 13 years to 75 years (standard deviation [s.d.]: 10.34). The greatest proportion of the patients were single (*n* = 198; 89.56%), unemployed (*n* = 103; 46.19%) and had no comorbidities (*n* = 171; 76.68%). Among those who did have comorbidities, the most common were HIV (*n* = 19; 8.52%), borderline personality disorder (*n* = 7; 3.14%) and major depressive disorder (*n* = 6; 2.26%). Only 10 (4.48%) were multi-morbid. Further details are provided in Table 1.

**TABLE 1:** Socio-demographic characteristics of participants.

Variables	Frequency (*n*)	%
**Age (years)**		
< 18	71	31.84
18–24	62	27.80
25–40	73	32.74
> 40	17	7.62
**Sex (*n* = 223)**		
Female	159	71.30
Male	64	28.70
**Marital status (*n* = 221)**		
Single	198	89.59
Married	19	8.60
Divorced	3	1.43
Widowed	1	0.45
**Occupation (*n* = 223)**		
Employed	28	12.56
Unemployed	103	46.19
School learners and students	85	38.12
Self-employed	4	1.79
Unknown	2	0.90
Pensioner	1	0.45
**Highest level of education (*n* = 223)**		
Primary school	40	17.9
High school	19	8.51
Tertiary	2	1.01
Unknown	162	72.6
**Comorbidities (*n* = 223)**		
Borderline personality disorder	7	3.14
Hypertension	5	2.24
Major depressive disorder	6	2.26
Other	5	2.24
HIV	19	8.52
Multimorbidity	10	4.48
None	171	76.68

Note: Age (years): mean = 24.6; minimum = 13; maximum = 75; standard deviation = 10.34.

HIV, human immunodeficiency virus.

### Clinical profile

The most common method of self-harm are overdosing and poisoning (*n* = 39 220; 98.65%). Attempts occurred most frequently during October (*n* = 39; 17.65), August (*n* = 29; 13.12) and February (21; 9.50%). A total of 21 participants (24.13%) reported that this was the second attempt or act and 9 (10.35%) reported that this was their third attempt or act. The most cited reasons for the current act were interpersonal, with family conflict (*n* = 69; 30.80%) and relationship problems (*n* = 61; 27.23%) as the most frequent reasons. More details are presented in Table 2.

**TABLE 2:** Clinical profile.

Variables	Frequency (*n*)	%
**Method classification (*n* = 223)**		
Bodily (or physical) harm	3	1.35
Overdose and poisoning	220	98.65
**Month of occurrence**		
January	19	8.60
February	21	9.50
March	13	5.88
April	17	7.69
May	12	5.43
June	15	6.79
July	11	4.97
August	29	13.12
September	10	4.52
October	39	17.65
November	17	7.69
December	19	8.59
**History of previous attempt (*n* = 87)**		
No	57	65.52
Yes	30	34.48
**Number of attempts (*n* = 87)**		
First attempt	57	65.52
Second attempt	21	24.13
More than twice	9	10.35
**Reason**		
Bullying	6	2.68
Grief	6	2.68
Financial issues	7	3.13
Family conflict	69	30.80
Relationship problems	61	27.23
Stress	13	5.80
Trauma	8	3.87
Other (e.g. employment-related issues, substance abuse, hopelessness, stagnancy etc.)	25	11.16
Multiple reasons	26	11.61

### Methods of self-harm

Self-poisoning and overdosing (*n* = 220; 98.65%) were the most predominate method used in this sample, with one laceration (*n* = 1; 0.45%) and two hangings attempted (*n* = 2; 0.90%). The use of prescription medication (*n* = 62; 27.80%), followed by OTC medication (*n* = 54; 24.22) were the most used techniques. Paracetamol was the most frequently used OTC medication, and ARV’s (*n* = 27; 27.27) were the most used prescription medication. Other less frequently used techniques included the use of corrosive agents (*n* = 4; 17.39%), cosmetic products (*n* = 3; 15.79%) and paraffin (*n* = 3; 15.79%) as shown in Table 3.

**TABLE 3:** Methods of self-harming.

Variables	Frequency (*n*)	%
**Method used**		
**Bodily harm**	3	1.**35**
Laceration	1	0.45
Hanging	2	0.90
**Overdose and poisoning**	220	98.65
Over-the-counter medication only	54	24.22
Prescription medication only	62	27.80
Both (OTC + prescription medication)	21	9.42
Pesticides only	45	20.18
Unknown or other substances only	19	8.52
Combination of other methods	19	8.52
**Breakdown of specific methods**		
**Over-the-counter medication**	101	-
Aspirin or Disprin	4	3.96
Paracetamol	40	39.61
Ibuprofen	14	13.86
Vitamins	14	13.86
Other or unspecified medication	29	28.71
**Prescription medication**	99	-
Analgesic medication	5	4.90
Antibiotics	9	9.09
Anti-cholesterol	2	2.02
Anti-depressant	9	9.09
Antihistamine	1	1.01
Anti-hypertensive	14	14.14
Anti-psychotics	6	6.06
ARVs	27	27.27
Benzodiazepines	7	7.07
Diabetic medication	5	5.21
Other or unspecified medication	10	10.10
Tuberculosis medication	4	4.04
**Pesticides**	45	-
Organophosphate pesticides	20	-
Rat poison	25	-
**Other substances**	19	-
Corrosive agents	4	17.39
Cosmetic products	3	15.79
Oil ingestion	2	10.53
Paraffin	3	15.79
Sanitiser	2	10.53
Other	5	17.24

OTC, over-the-counter.

### Patterns and associations of self-harming by age

Pearson Chi-square test of association was used to identify the factors that were significantly associated with age above or below 18 years. Increased frequency of self-harming for both ages was significantly associated with females (*p* = 0.000). Additionally, comorbidities (*p* = 0.001) were significantly associated with the over 18 years group. The use of OTC medication was significantly associated with younger participants whereas the use of prescription medication and pesticides was significantly associated with older age (*p* = 0.041). A significant association between age and reason for self-harming was identified (*p* = 0.000) with family conflict significantly associated with younger patients and relationship problems among the older patients. More details to be found in Table 4.

**TABLE 4:** Bivariate analysis stratified by age.

Variables	*n*	%	Age				Chi^2^	*p*
			≤ 18 years		˃ 18 years			
			*n*	%	*n*	%		
**Sex**	-	-	-	-	-	-	23.4862	0.000*
Female	159	71.30	78	86.99	81	59.56	-	-
Male	64	28.70	9	10.34	55	40.44	-	-
**Comorbidities**	-	-	-	-	-	-	22.1919	0.001*
Borderline personality disorder	7	13.46	1	1.15	6	4.41	-	-
Hypertension	5	9.62	1	1.15	4	2.94	-	-
Major depressive disorder	6	11.54	0	0.00	6	4.41	-	-
Other	5	9.62	1	1.15	4	2.94	-	-
Retroviral diseases	19	36.54	2	2.30	17	12.50	-	-
Multimorbidity	10	19.23	1	1.15	9	6.62	-	-
None	171	76.68	81	93.10	90	66.18	-	-
**Specific method used**	-	-	-	-	-	-	13.1180	0.041*
Bodily harm	3	1.35	0	0.00	3	2.21	-	-
Over-the-counter medication only	54	24.22	27	31.03	27	19.85	-	-
Prescription medication only	62	27.80	26	29.89	36	26.47	-	-
Both (OTC + prescription medication)	21	9.42	7	8.05	14	10.29	-	-
Pesticides only	45	20.18	9	10.34	36	24.67	-	-
Unknown or other substances only	19	8.52	9	10.34	10	7.35	-	-
Combination of other methods	19	8.52	9	10.34	10	7.35	-	-
**Reason**	-	-	-	-	-	-	73.6279	0.000*
Bullying	6	2.68	6	6.90	0	0.00	-	-
Grief	6	2.68	3	3.45	3	2.21	-	-
Financial issues	7	3.13	2	2.30	5	3.68	-	-
Family conflict	69	30.80	48	55.17	21	15.44	-	-
Relationship problems	61	27.23	6	6.90	55	40.44	-	-
Stress	13	5.80	1	1.15	12	8.82	-	-
Trauma	8	3.87	5	5.75	3	2.21	-	-
Other	25	11.16	4	4.60	20	13.76	-	-
Multiple reasons	26	11.61	12	13.79	14	10.29	-	-

OTC, over-the-counter.

The female gender (*p* = 0.000) showed 6.6 greater odds for parasuicide as compared to males. Younger participants who reported family conflict and older participants experiencing relationship problems were 1.3 times more likely to self-harm themselves than those who gave other reasons, as presented in Table 5.

**TABLE 5:** Multivariate logistic regression by age.

Variables	Odds ratio	Standard error	*P* > |*z*|	95% CI
Marital status	2.5474140	1.1474670	0.038	1.0536130, 6.159109
Occupation	0.9302905	0.1413176	0.634	0.6907409, 1.252916
Comorbidities	1.2180600	0.2597180	0.355	0.8019982, 1.849968
Specific method used	1.2846770	0.1576494	0.041	1.0100400, 1.633989
Reason for attempt	1.0872890	0.0941605	0.334	0.9175504, 1.288427

CI, confidence interval.

### Patterns and associations of parasuicide stratified by sex

Marital status (*p* = 0.057) and occupation (*p* = 0.000) were significantly associated with self-harming behaviour with unmarried female students and unmarried unemployed males having a higher likelihood of self-harming behavioural acts. The use of prescription medication was significantly associated with female patients whereas for males, it was the use of pesticides (*p* = 0.000). Family conflict was more significantly associated with females and relationship problems were associated significantly with male patients (*p* = 0.008).

Marital status and method used were the only two factors that remained significant during multivariate logistic regression. Unmarried participants in both sexes were 2.5 times more likely to show self-harming behaviour. Additionally, males had 1.3 greater likelihood of using pesticides and females had similar odds for the use of prescription medication.

## Discussion

Self-harming behaviour was observed across different age groups and was most prominent in those aged 16–25 years with a sample mean age of 24.6 years. The youngest participant was 13 years and the oldest was 75 years (s.d.: 10.34) (Table 1). This study’s findings are consistent with those of another South African study conducted in the KwaZulu-Natal province in 2017,^7^ and at the nearby Brits Hospital.^6,^ This contrasts with the Zimbabwean study,^6^ where most participants were between the ages of 31 years and 35 years.

The study aligns with other South African studies in finding that females are more vulnerable (Table 1).

There is a significant correlation between self-harming behaviour (or act) and marital status, with single people being more likely to engage in such action (Table 1). Most of the patients in this study were single (*n* = 198; 89.59%). A multivariate logistic model found that unmarried people of both genders were 2.5 times more likely to conduct self-harming acts. This is consistent with the findings of a systematic review and meta-analysis study conducted in South Asia, which found that single persons commit more self-harming acts than married people.^18^ The current study’s findings are compatible with the findings in KwaZulu-Natal^4^ that showed that single persons have a greater self-harming risk than married people. According to statistics, 74% of those who reported self-harming behaviour were single, with only 16% married.^7^ In Brits, North west Province, South Africa, it was found that 74% of people were single.

This study found a robust link between self-harming behaviour and occupation (*p* = 0.000), with unmarried unemployed males being at a higher risk. The unemployment rate in this study is 46.19% (103 participants), whereas the employment rate is 12.56% (28 people). Economic issues play a significant part in the prevalence of self-harming behaviour in Zimbabwe; in Cape Town, depression is more connected with self-harming behaviour than a variety of proximal and distal economic factors among young males living in South Africa’s endemic poverty. This has substantial public health implications, underlining the importance of increasing young men’s access to psychiatric therapies, with depression screening playing a critical role. In Limpopo province of South Africa, insufficient resources and lack of aid are the key reasons associated with self-harm. The stated causes of self-harming behaviour were characterised as ‘bullying’, ‘grief’, ‘financial issues’, ‘family conflicts’, ‘relationship problems’, ‘stress’, ‘trauma’, ‘multiple reasons’ and ‘other’. The most common causes were family conflicts (*n* = 69; 30.80%), followed by relationship issues (*n* = 61; 27.23%). Most patients who cited family concerns as a motivation for self-harming behaviour (*n* = 69) were males (*n* = 48; 55.17%), whereas the majority of those citing relationship problems as a motive were females (*n* = 55; 40.44).

The self-harming method was recorded, which included ‘hanging’, ‘ingestion poisoning’, ‘prescription’ or ‘OTC’ drug overdose and ‘laceration’ injury. The most common tactics in this study were overdose and self-poisoning (*n* = 220; 98.65%), followed by two hanging attempts (*n* = 2; 0.90%) and one laceration (*n* = 1; 0.45%). This is consistent with the findings of a South African study published in 2009^9^ which found that self-poisoning was the most reported technique (80.3%), and another study conducted in Istanbul, Turkey,^16^ which found 94.4% drug intoxication and 1.4% self-harm with sharp items.

This study found that October had the highest rate of self-harming cases (*n* = 39; 17.65%), coinciding with South Africa’s spring season. Interestingly, the lowest rates were reported in September (*n* = 10; 4.52%), while at the nearby Brits Hospital, higher rates of self-harming behaviour were observed in January and February.

## Conclusion

Self-harm is a common behaviour among single females under 40 years, often resulting from conflict in romantic and family relationships. Age and sex play a significant role in the risk of the behaviour. Younger females with family conflict are more likely to use OTC medication, while older females with retroviral diseases are more likely to use prescription medication. Males are more likely to use pesticides. Further research, including preventative strategies, may shed further information on the management of behaviour.
